# Advancements in Cardiovascular Imaging: Substituting Magnetic Resonance Imaging for Contrast-Enhanced Computed Tomography in the Assessment of Patent Ductus Arteriosus

**DOI:** 10.7759/cureus.72231

**Published:** 2024-10-23

**Authors:** Toru Miyoshi, Fuminori Shinozuka, Toshio Honda, Yusuke Akazawa, Osamu Yamaguchi

**Affiliations:** 1 Department of Cardiology, Pulmonology, Hypertension, and Nephrology, Graduate School of Medicine, Ehime University, Toon City, JPN; 2 Department of Radiology, Sadamoto Hospital, Matsuyama, JPN; 3 Department of Cardiology, Sadamoto Hospital, Matsuyama, JPN

**Keywords:** adult congenital heart disease (achd), black blood imaging, heart failure, magnetic resonance imaging, patent ductus arteriosus closure

## Abstract

A male in his seventies was admitted for a patent ductus arteriosus (PDA) closure. We planned a catheter-based closure but avoided pre-procedural contrast-enhanced CT due to his low estimated glomerular filtration rate. Instead, we used magnetic resonance imaging (MRI) (Philips, 3T, Ingenia Elition, Philips Healthcare, Amsterdam, The Netherlands). An MRI of the aorta showed that the PDA was aneurysmal (Krichenko classification, type D). The Qs/Qp ratio via two-dimensional phase-contrast MRI was 1.78. Black blood imaging was used to obtain accurate diameter determination. Black Blood Imaging and aortography did not differ in any dimension. The procedure was completed using an Amplatzer Vascular Plug II (Abbott, Abbott Park, IL) 16 × 12 mm, achieving PDA closure without complications, and the patient was discharged. MRI provided an accurate assessment of the vascular morphology using non-contrast-enhanced black blood imaging, suggesting a gradual change in PDA morphology.

## Introduction

The ductus arteriosus originates from the distal left sixth arch embryologically, connecting the aortic arch and the proximal left pulmonary artery. Normally, it closes within one to two days after birth, but if it remains open, it results in a patent ductus arteriosus (PDA). This left-to-right shunt condition increases pulmonary blood flow and imposes a volume load on the left heart [1]. In adults, PDA rarely coexists with other congenital heart defects. With aging and arteriosclerosis, atherosclerotic changes and calcification can be observed in adults' ductus arteriosus and the aorta [2,3]. In addition, PDA can cause arterial endocarditis. The annual incidence of infective endocarditis (IE) associated with PDA is reported to be 0.14% [4] (95% binomial confidence interval [CI]: 0.05%-0.5%). Because of the high-flow nature of PDAs, the resulting elevated pressure gradient renders the intimal layer more vulnerable to damage, thereby increasing the risk of colonization by pathogenic microorganisms, which may subsequently adhere to the site. Small ductus arteriosus with no evidence of volume overload have a low incidence of arterial endocarditis, making the closure indication for elderly patients unclear. However, recent advancements in transcatheter closure techniques have enabled the use of multiple devices. This procedure is particularly effective in cases with left-to-right shunting. Adequate preoperative evaluation is essential for performing transcatheter closure. Although ECG-gated CT scan is effective, chronic kidney disease is not uncommon among elderly patients. Therefore, we conducted preoperative examinations using MRI, compared the imaging with previous scans to detect morphological changes in PDA, and reported a case where this approach was useful in device selection for transcatheter closure.

## Case presentation

**Figure 1:**
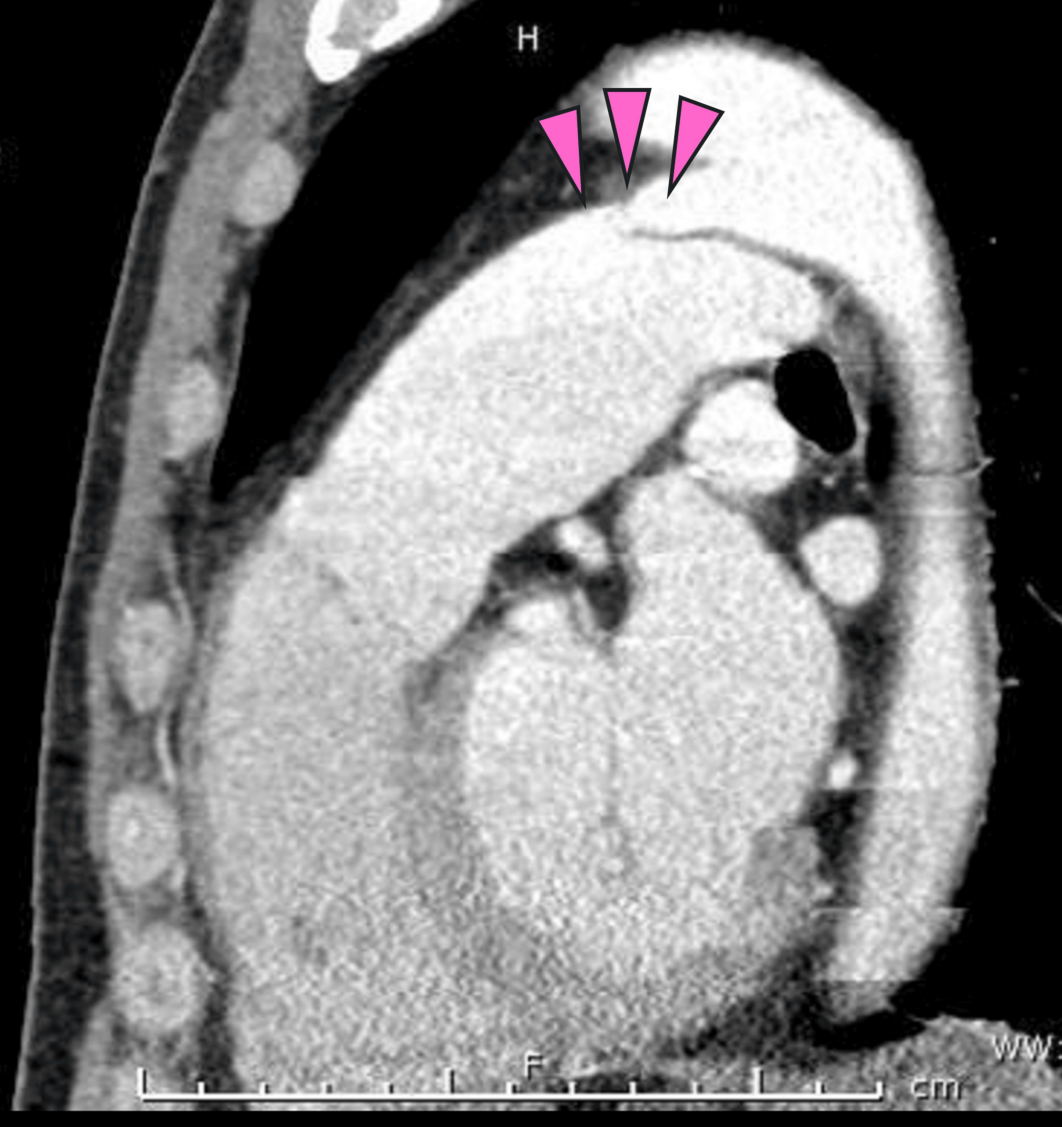
Contrast-enhanced computed tomography taken 17 years ago.

A 76-year-old male patient was diagnosed with a PDA 17 years ago, classified as Krichenko type A by contrast computed tomography (CT; Figure 1).

**Figure 2:**
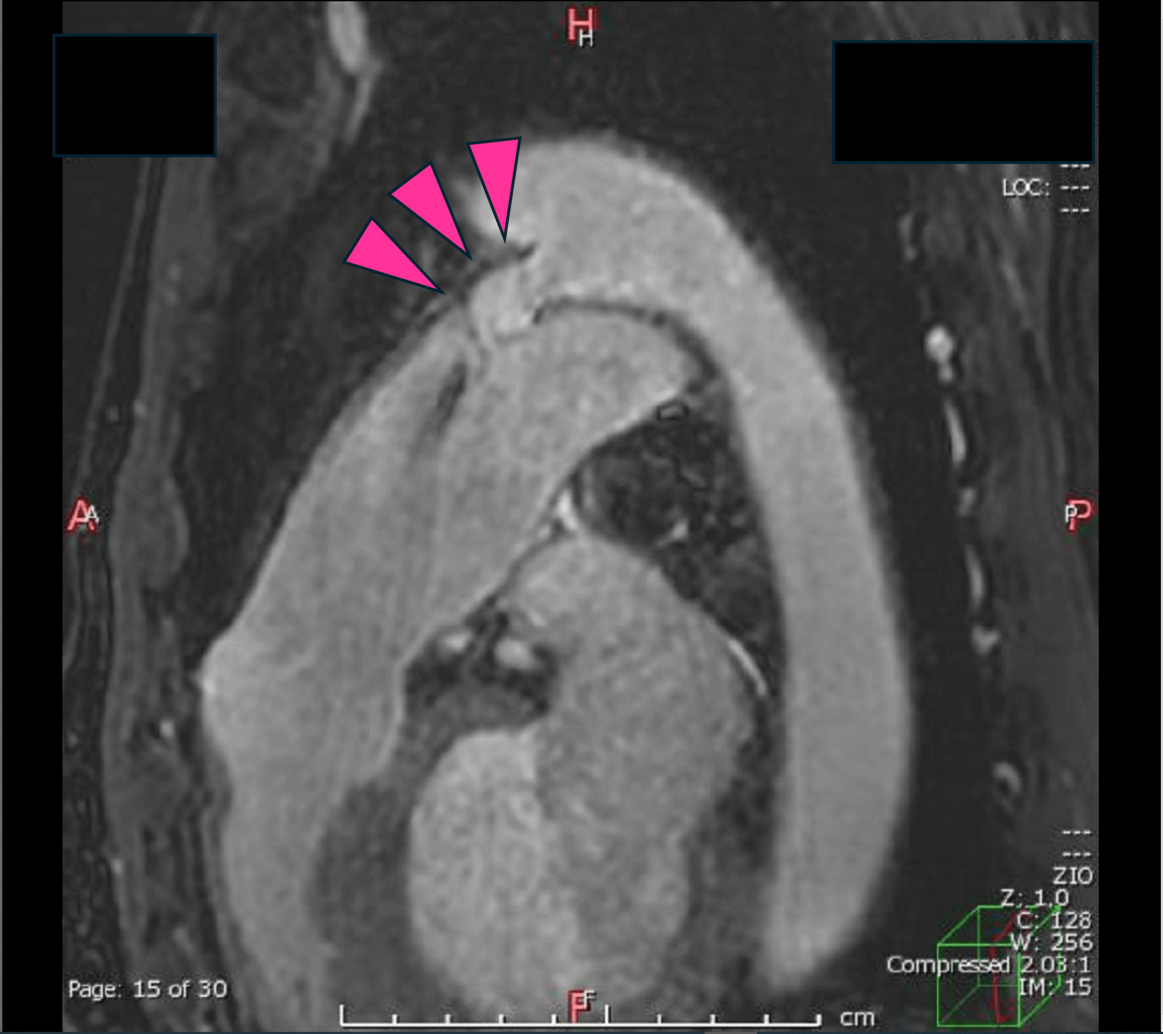
Magnetic resonance imaging of the aorta revealed an aneurysmal patent ductus arteriosus (Krichenko classification, type D).

His condition remained stable until five years ago when significant left ventricular enlargement was observed. His B-type natriuretic peptide (BNP) and tricuspid regurgitation pressure gradient (TRPG) levels were elevated in the spring of this year, and he was managed with medication. His right heart catheterization results were as follows: Pulmonary artery pressure was 32/12 (22) mmHg, pulmonary artery wedge pressure was 10 mmHg, and right atrial pressure was 8 mmHg when blood pressure was 152/54 (90 mmHg). Despite these results, his exertional dyspnea worsened, prompting the decision to perform catheter-based closure of the PDA. With an estimated glomerular filtration rate of 28 mL/min/1.73 m², it was preferable to avoid pre-procedural contrast-enhanced CT. Consequently, magnetic resonance imaging (Philips, 3T, Ingenia Elition, Amsterdam, The Netherlands) was employed as an alternative. MRI imaging of the aorta revealed an aneurysmal PDA (Krichenko classification, type D; Figure 2).

**Figure 3:**
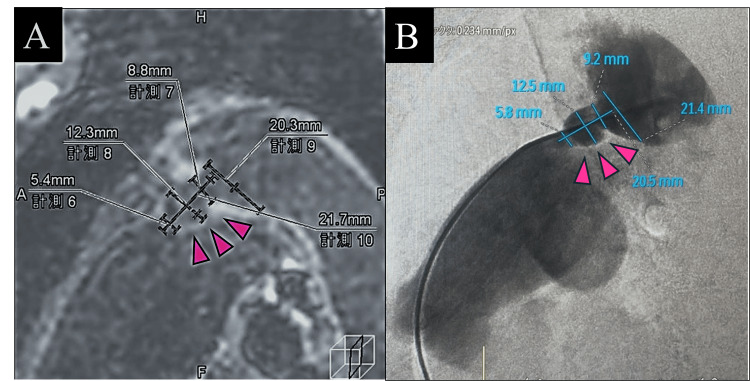
Black blood imaging and arteriography. (A) Measurements made using Black blood imaging; (B) measurements performed using arteriography.

It was found that the morphology of the PDA had significantly changed compared to the imaging performed 17 years ago. Additionally, the Qs/Qp ratio calculated via two-dimensional phase-contrast MRI was 1.78. For precise diameter determination before closure, black blood imaging was utilized (Figure 3).

**Figure 4:**
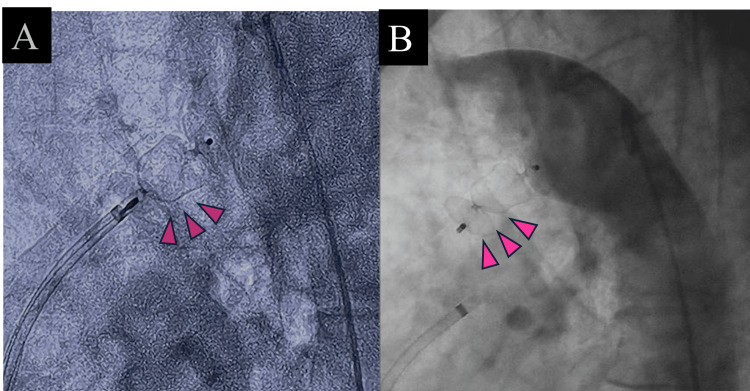
Patent ductus arteriosus closure. (A) Amplatzer Vascular Plug II (Abbott) 16 mm × 12 mm; (B) the PDA disappeared completely with the plug. PDA, patent ductus arteriosus

The findings from MR arteriovenography were also concordant. The procedure was performed using a 16 mm × 12 mm Amplatzer Vascular Plug II (Abbott, Abbott Park, IL), achieving closure of the PDA without complications (Figure 4).

PDA closure was performed after adequate preoperative saline rehydration. For PDA closure, 100 cc of contrast agent was required. The postoperative estimated glomerular filtration rate (eGFR) was 32 mL/min/1.73 m^2^, and there was no worsening of renal dysfunction. The patient was subsequently discharged.

## Discussion

PDA is typically treated in childhood through catheter closure procedures or surgical interventions, both of which have demonstrated a favorable long-term prognosis [5-7]. Currently, therapeutic options for PDA include catheter-based techniques such as the deployment of coils, the Amplatzer Duct Occluder, and vascular plugs, as well as surgical approaches like ligation and division. The surgical closure of a PDA in adults is more complex than the simple ligation often performed in childhood; it requires open-heart surgery with cardiopulmonary bypass. As a result, catheter-based treatments are valuable for adult patients.

In the follow-up of patients with PDA, echocardiography remains the easiest and least invasive method. Although it is challenging to evaluate morphological changes in PDA, it is feasible to monitor the progression of right ventricular overload. Additionally, when there is a progressive increase in right ventricular overload, catheterization becomes valuable in guiding treatment decisions. For PDA, calculating the Qs/Qp ratio, rather than Qp/Qs, provides more relevant information for determining the therapeutic approach. If PDA closure is deemed necessary, a thorough assessment of the ductal morphology is crucial. 

 Although PDA can now be closed using catheterization, it remains crucial to accurately evaluate its morphology and size before the procedure. Contrast-enhanced CT is widely considered the gold standard for this evaluation. However, in cases where the use of iodinated contrast agents must be minimized, such as in patients with chronic kidney disease, MRI serves as a valuable alternative. While imaging of the descending aorta using MRI can be challenging, the integration of electrocardiogram (ECG) gating allows for the acquisition of stable images. In some instances, PDA may present with calcification, which can impede accurate assessment of its morphology with standard magnetic resonance angiography (MRA). Consequently, we performed black-blood imaging to evaluate the morphology of the descending aorta and ductus arteriosus [8]. This technique, commonly used in neurosurgery to assess vertebral arteries, was also beneficial in our case. It enabled precise determination of the ductus arteriosus's morphology and size, thereby facilitating catheter-based treatment.

Furthermore, in this case, the morphology of the PDA had changed from Krichenko classification type A to type D, an observation that has not been widely reported in the literature and suggests that the morphology of PDA may change over time. Although it is impractical to perform repeated contrast-enhanced CT scans, MRI offers a more *patient-friendly* alternative due to its lack of radiation exposure and contrast agent effects. Once PDA is identified, conducting MRI examinations every three to five years allows for the monitoring of morphological changes and the Qs/Qp ratio, which would be useful for determining subsequent treatment strategies. After PDA closure, follow-up using echocardiography is recommended.

## Conclusions

We reported a case in which MRI was used instead of contrast-enhanced CT before catheterization for PDA, allowing for adequate evaluation. This imaging modality would be useful not only in determining which device to close but also for follow-up over time. In addition to regular MRA, an additional black blood scan is a useful method to ensure that the appearance of the vessels is captured.
